# Orbital tuberculosis in childhood with intracranial extension: a case report

**DOI:** 10.1186/1757-1626-3-38

**Published:** 2010-01-28

**Authors:** Navneet Tuli

**Affiliations:** 1 Department of Ophthalmology, Himalayan Institute of Medical Sciences, HIHT University, Swami Ram Nagar, Dehradun 248140, India

## Abstract

The common causes of orbital masses in pediatric age group include pseudotumour, lymphomas, hemangioma and dermoid cyst. Orbital tuberculosis is rare especially in children. We report a case of 5 year old child who presented with proptosis and gross diminution of vision due to orbital tuberculoma. Ocular examination of the left eye revealed proptosis with the eyeball displaced downwards and forwards. Vision was counting finger close to face. CT Scan showed an extraconal soft tissue mass along posteromedial side of left orbit with lateral displacement of medial rectus muscle. On antitubercular treatment, proptosis regressed and visual recovery was observed over a period of six month vision, in the left eye at the last followup was 20/30.

## Case presentation

A 5 year old Indian boy was admitted in April 2007 for investigation of proptosis in his left eye. He complained of fever and swelling in the neck for 2 months and protuberance of his left eye for one month. There was no significant family history. The child had not been immunized.

The general physical examination showed the child to be pyrexial and pale. The left preauricular, submental and cervical lymph nodes were enlarged. Systemic examination was unremarkable.

**Figure 1:**
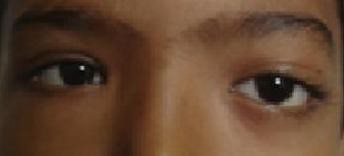
**Showing proptosis left eye with eyeball displaced downwards and forwards**.

Ocular examination of the left eye revealed proptosis with the eyeball displaced downwards and forwards (Figure. 1). The extraocular movements of left eye showed limitation of adduction, dextro-elevation and dextro-depression. Vision was counting finger close to face. Relative afferent pupillary defect was present in the left eye but the optic disc and macula were normal and periphery was unremarkable. Fundoscopy was within normal limits. The right eye was essentially normal.

**Figure 2:**
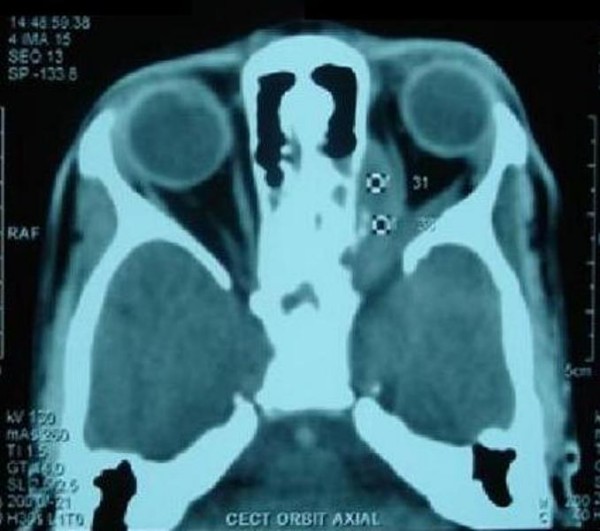
**Axial CT showing mass superiorly up to superior orbital fissure with widening of fissure**.

**Figure 3:**
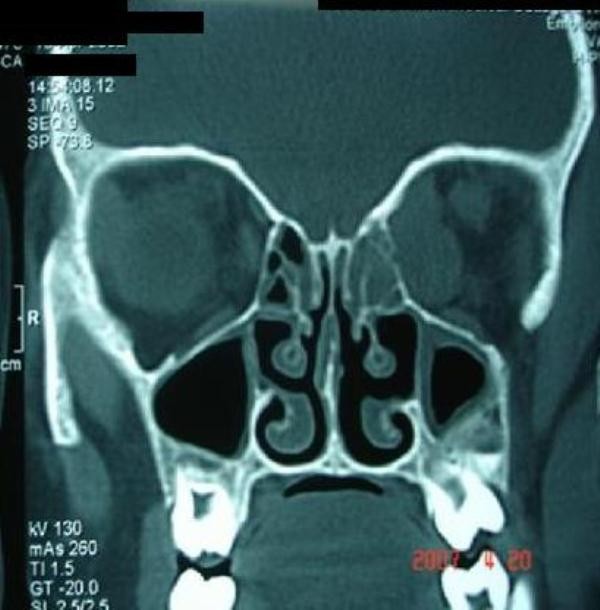
**Coronal CT scan showing mass extending inferiorly to infratemporal fossa with bony erosion**.

Investigations included Hb 9.4 gm%, leucocytes 9800/cmm, erythrocyte sedimentation rate (ESR) 25 mm in first hour. The chest radiograph was normal. Orbital computed tomography (CT) Scan showed an extraconal soft tissue mass along posteromedial side of left orbit with lateral displacement of medial rectus muscle. The mass was extending inferiorly to infratemporal fossa with signs of bony erosion and superiorly up to superior orbital fissure causing widening of fissure (Figure 2, Figure 3). Mantoux test was positive showing induration and erythema of 15 × 15 mm in diameter. HIV was non-reactive.

Fine needle aspiration cytology (FNAC) of the cervical lymph nodes showed inflammatory granulomatous lesion with caseation suggestive of tuberculosis. Medial orbitotomy and incisional biopsy of mass was performed which revealed similar features. Cultures for acid fast bacilli were negative. Clinical diagnosis of tuberculosis was made on the basis of strongly positive Montoux test and the caseating granulomatous lesion seen in histopathology. The patient was started on antitubercular therapy consisting of isoniazid, rifampicin, pyrazinamide and ethambutol for initial 60 days, followed by isoniazid and riampicin for 10 months.

**Figure 4:**
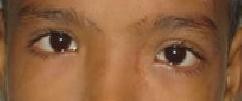
**After antitubercular treatment**.

On antitubercular treatment, proptosis regressed and ocular motility of left eye improved. The extra-ocular movements were full in the last follow-up (Figure 4) and visual recovery was observed over a period of six month. The final visual acuity was 20/30.

## Discussion

Tubercular involvement of the orbit is uncommon. It may occur as a result of hematogenous spread of infection from a distant site or by direct extension of infection from adjacent sinus, the lacrimal gland or sac [1]. It is usually unilateral [2]. Orbital involvement may cause proptosis, dacryoadenitis, sinus formation, keratitis and ectropion [3].

To establish the diagnosis of orbital tuberculosis, evidence of systemic active or inactive tuberculosis is looked for. The pulmonary loci may not be evident clinically or radiologically. Orbital tuberculosis has been reported in individuals who did not suffer from pulmonary tuberculosis but associated tuberculosis in some other areas like tubercular sinusitis and constrictive pericarditis [4]. The diagnosis is usually based on positive tuberculin test, caseating granulomatous inflammatory lesions on histopathological examination of tissue and positive culture of mycobacterium tuberculosis if specimen are obtained early in the course of the disease. Acid fast bacilli are difficult to detect in the pathological specimen.

In our case, the chest radiograph did not show presence of primary complex. However, the patient had cervical lymphoadinitis. Montoux test was strongly positive and typical features of tuberculosis were seen on histopathology. The disease completely resolved with antitubercular therapy.

The present case emphasizes that in endemic areas, tuberculosis should be considered as an etiology in the evaluation of childhood proptosis. It highlights the fact that awareness of many faces of tuberculosis is important for an ophthalmologist.

## Abbreviations

FNAC: Fine needle aspiration cytology.

## Competing interests

The author declares that they have no competing interests.

## Consent

Written informed consent was obtained from the patient for publication of this case report and accompanying images. A copy of the written consent is available for review by the journal's Editor-in-Chief.
